# Analysis of risk factors and establishment of predictive models for neonatal necrotizing enterocolitis: a retrospective study

**DOI:** 10.1186/s13052-025-01930-y

**Published:** 2025-03-14

**Authors:** Keqin Liu, Jinjin Guo, Yaqi Zhu, Jixin Yang, Yanwei Su

**Affiliations:** 1 Department of Nursing, Tongji Hospital, Tongji Medical College, Huazhong University of Science and Technology, Wuhan, 430030 Hubei China; 2 School of Nursing, Tongji Medical College, Huazhong University of Science and Technology, Wuhan, 430030 Hubei China; 3 Department of Pediatric Surgery, Tongji Hospital, Tongji Medical College, Huazhong University of Science and Technology, Wuhan, 430030 Hubei China

**Keywords:** Necrotizing Enterocolitis, Risk factors, Human milk proportion, Small gestation age, Neonatal sepsis

## Abstract

**Background:**

Necrotizing enterocolitis (NEC) is a leading gastrointestinal condition in preterm infants, characterized by significant morbidity and mortality. Early recognition of risk factors is crucial for its prevention and prediction. This study focuses on identifying factors that contribute to the development of NEC in neonates.

**Methods:**

A case-control study that looked back at 144 newborns hospitalized to a Wuhan hospital between January 2010 and March 2023 for NEC was carried out. Over the same period, another 144 children without NEC were identified and selected as the non-NEC group for comparison, following a 1:1 pairing ratio. The relevant data from these two groups of newborns were compared. Univariate analysis was conducted using T-tests or χ^2^ tests, followed by multivariate logistic regression to determine independent risk factors and develop a clinical prediction model.

**Results:**

A total of 288 neonates (144 NEC and 144 non-NEC) were enrolled. The independent risk variables for NEC, as shown by the multivariate logistic regression analysis (*p* < 0.05), were Small for Gestational Age (SGA), neonatal sepsis, neonatal hyperbilirubinemia, and non-human milk (HM) feeding. Furthermore, ROC (receiver operating characteristic) analysis showed that the AUC (area under the curve) of the Logistic regression model predicting the effect of neonatal necrotizing enterocolitis was 0.746, suggesting a high level of discriminative ability in differentiating efficacy. This model can be instrumental in facilitating early identification of infants prone to developing NEC in clinical settings.

**Conclusion:**

In conclusion, the risk factors associated with newborn NEC include SGA, neonatal sepsis, and non-HM feeding. Newborn hyperbilirubinemia may potentially serve as a protective factor against NEC.

**Supplementary Information:**

The online version contains supplementary material available at 10.1186/s13052-025-01930-y.

## Introduction

Necrotizing enterocolitis (NEC) is a grave disorder that mostly affects premature newborns and is the primary cause of mortality resulting from gastrointestinal disease in this susceptible population [1]. Even with the progress in contemporary medicine, NEC continues to have high morbidity rates of 7-13% and mortality rates of 20-30%, particularly affecting preterm infants, those with low birth weight (LBW), and neonates receiving enteral nutrition [2–4]. NEC is characterized by a disruption of intestinal mucosal integrity. When inflammation and injury are limited, medical treatment can be effective. However, if the condition progresses to necrosis and bowel perforation, surgical intervention becomes necessary [5]. The mortality rate for extremely LBW neonates undergoing surgery for NEC can be as high as 20–50%. Survivors may face significant complications, including neurodevelopmental impairments, short-bowel syndrome, and growth restriction, with limited new therapies available [6]. Over the past decade, mortality rates and treatment approaches have largely remained unchanged, despite ongoing improvements in the care of the smallest preterm infants. Moreover, even with advances in neonatal intensive care, high morbidity rates persist among infants with NEC in high income countries [7, 8].

The pathogenesis of NEC remains unclear, but it is widely recognized as a multifactorial disease [9, 10]. The prevailing view is that NEC is primarily associated with factors such as intestinal immaturity, infections, genetic predisposition, dysbiosis, and mucosal immune dysfunction [11]. Considered as main risk factors for NEC development include LBW and preterm delivery [12]. However, the pathogenesis of NEC is very complex, involving maternal, and neonatal treatment, etc., and it is impossible to predict the risk of the NEC by using a single factor. The disease’s onset is often subtle and progresses rapidly, making early detection challenging. Initial symptoms like feeding intolerance or abdominal distention can quickly escalate to severe complications, including sepsis, disseminated intravascular coagulation, and death [13, 14]. Therefore, early identification of risk variables connected to NEC is therefore very crucial for early diagnosis, early treatment, and prevention of NEC.

Retroactively examined were the clinical records of 144 non-NEC newborns admitted to Tongji Hospital, Tongji Medical College, Huazhong University of Science and Technology between January 2010 and March 2023 and 144 neonates with NEC. This project is to investigate NEC-related risk variables and build a prediction model to find early-stage preterm children who could develop NEC, therefore providing a basis for early detection and management of NEC in premature infants.

## Materials and methods

Between January 2010 and March 2023, newborns hospitalized in the Neonatal Intensive Care Unit (NICU) at Tongji Hospital, Tongji Medical College, Huazhong University of Science and Technology, were included in this single-center, retroactive 1:1 case-control research. Approved by the Ethics Committee of Tongji Medical College, Huazhong University of Science and Technology (Number S1191), the research followed the Declaration of Helsinki.

### Population selection

Data collection was conducted through a review of the patient’s medical record. Neonates with confirmed NEC, classified in the database between January 2010 and March 2023, were identified based on modified Bell’s criteria. To minimize population heterogeneity, the study excluded congenital intestinal diseases (congenital megacolon, congenital colonic stenosis, short bowel syndrome, congenital ganglionated megacolon, congenital intestinal atresia, etc.) based on clinical cases. Meanwhile, neonates with hospital stays were less than 7 days, feeding was not initiated before the onset of NEC, death was within 2 weeks after hospitalization, and cases that were incomplete or unavailable were excluded. Finally, 144 NEC infants 144 met the study criteria. Ultimately, 144 NEC cases and 144 matched controls, who were hospitalized for other neonatal diseases during the same period, were selected from the database. Controls were matched by gender, date of birth (within six months), gestational age (GA: within one week), and birth weight (BW: within one hundred grams).

### Data collection

Data were taken from newborn medical records, imported into Microsoft Excel, and then examined with SPSS version 26.0 (IBM Corp, Armonk, NY). The collected data were categorized into four main areas: (i) demographic information, including date of birth, sex, GA, and BW; (ii) maternal-related factors, including mode of pregnancy, status of pregnancy, mode of delivery (cesarean section/vaginal delivery), antenatal corticosteroids use, and maternal complications (pregnancy-induced hypertension, gestational diabetes, premature rupture of membranes, intrauterine distress); (iii) neonatal factors, including birth status (BW, Apgar score, neonatal asphyxia, and classification as large, appropriate, or small for GA), and neonatal diseases before NEC (respiratory distress syndrome, neonatal sepsis, neonatal pneumonia, patent ductus arteriosus); and (iv) feeding related factors, including first time to feeding, feeding methods (breastfeeding, formula or mixed), HM proportion (exclusive formula feeding, low-HM feeding, and high-HM feeding), probiotics use. Infants were grouped based on the proportion of HM intake during hospitalization relative to their total enteral feeding (0%, < 50%, ≥ 50%). Infants with ≥ 50% HM intake were categorized into the high-HM group, while those with < 50% HM intake were categorized into the low-HM group. Infants receiving no HM or exclusively formula-fed were classified into the non-HM group. In our study, the probiotics used by the preterm infants we included were bifidobacterium and lactobacillus. Data were collected independently by two researchers using the electronic medical records. Discrepancies were discussed within pairs with a third reviewer if needed. For each case and control, data were gathered from birth until the day before NEC onset in the case and the corresponding control.

### Definition

NEC was defined according to the Bell staging criteria, with staging independently confirmed by two NEC specialists based on clinical records and abdominal radiographs. Small for gestational age (SGA) is described as having a BW that falls below the 10th percentile for GA. Neonatal sepsis was classified as a clinical syndrome with non-specific symptoms resulting from pathogenic infection. In this study, sepsis included early-onset (EOS) and late-onset (LOS) cases. EOS was defined as sepsis diagnosed within the first 72 h of life, while LOS referred to sepsis diagnosed in neonates with a pathogen detected in blood cultures taken 72 h or more after birth, with antibiotics administered for at least 5 days. Human milk (HM) feeding included both maternal milk and pasteurized donor milk. The assessment of HM intake was based on the percentage of HM in the total enteral feeding, the overall proportion of enteral intake, and the cumulative volume of HM before the beginning of NEC.

### Statistical analysis

The statistical analyses were performed using IBM SPSS software (version 26; Chicago, IL, USA). Continuous variables are shown as medians with interquartile ranges (IQR, Interquartile percentile) and were compared using the Mann-Whitney U test. Categorical variables were represented as counts and percentages and were evaluated using either the χ^2^ test or Fisher’s exact test. Adjusted odds ratios (ORs) were calculated and independent risk variables were identified using multivariate logistic regression. Variables that had a *p*-value less than 0.05 in the univariate analysis or were considered clinically relevant were included in the logistic regression models. The predictive value was assessed using a receiver operating characteristic (ROC) curve, and establish the risk prediction model. A *p*-value below 0.05 was deemed to be statistically significant.

## Results

**Fig. 1:**
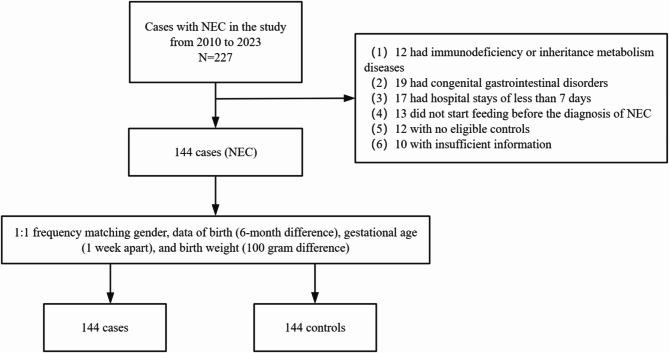
Flow chart of selection of the study population. Figure Legends NEC, necrotizing enterocolitis

During the research period, a total of 144 newborns diagnosed with NEC were discovered. Additionally, 144 control infants were selected based on their birth date, GA, and BW to match the NEC group. Figure 1 illustrates the selection process for these neonates. In both the NEC cohort and the control group, the gender proportions were the same, with males accounting for 59.03% and females for 40.97%. The median GA was 31.14 weeks (IQR 28.71–33.29) in the NEC group compared to 30.93 weeks (IQR 29.00-33.39) in the control group. The median BW in the NEC group was 1415 g (IQR 1145–1820), whereas in the control group, it was 1420 g (IQR 1162–1797). There were no significant differences in birth weight between the two groups. Table 1 provides a comprehensive overview of the clinical features of both groups.

**Table 1:** Demographic characteristics of the study cohort

Neonatal demographics	Control (*n* = 144)	NEC (*n* = 144)	Test (c^2^/Z)	*P*
Gender				
Male	85(59.03%)	85(59.03%)	0.000	1
Female	59(40.97%)	59(40.97%)		
Gestational age (w)	30.93(29.00-33.39)	31.14(28.71–33.29)	-0.621	0.535
Birth weight (g)	1420(1162–1797)	1415(1145–1820)	-0.064	0.949

There was no significant difference between the control and NEC groups

Abbreviations: NEC, Necrotizing enterocolitis

Table 2 shows the comparison of maternal, fetal, and feeding factors between NEC and control neonates. It is important to mention that neonatal and feeding factors are substantially connected with the probability of having NEC (*p* < 0.05). The rate of SGA was 30.6% (44 out of 144) in the NEC group, whereas it was 14.6% (21 out of 144) in the control group. This difference was statistically significant before NEC onset (*p* = 0.001). In addition, the NEC group exhibited lower median Apgar scores at one minute (7 [IQR 6–7]) than the control group (7 [IQR 6–8]). Neonatal sepsis rates were higher in the NEC group [45 (31.3%) vs. 11 (7.6%), *p* < 0.001]. Based on human milk intake, infants were categorized into three groups: high human milk feeding (≥ 50%, 83 infants), low human milk feeding (< 50%, 37 infants), and exclusive formula feeding (168 infants). The HM proportion significantly differed between the NEC and control groups (*p* < 0.05).

**Table 2:** Comparison of maternal, fetal, and feeding factors between NEC and control neonates

Variables	Control (*n* = 144)	NEC (*n* = 144)	χ^2^/Z	*P*
**Maternal factors**				
In vitro fertilization	48 (33.30%)	48 (33.30%)	0.000	1.000
Multiple fetus	56 (38.90%)	61 (42.40%)	0.360	0.549
Cesarean section	111 (77.10%)	99 (68.80%)	2.532	0.112
Antenatal corticosteroids	100 (69.40%)	104 (72.20%)	0.269	0.604
Premature rupture of membranes	43 (29.90%)	34 (23.60%)	1.436	0.231
Intrauterine distress	18 (12.50%)	19 (13.20%)	0.031	0.860
GDM	34 (23.60%)	34 (23.60%)	0.000	1.000
Pregnancy-induced hypertension	42 (29.20%)	35 (24.30%)	0.869	0.351
**Neonatal factors**				
SGA	21 (14.6%)	44 (30.6%)	10.511	0.001
LBW infant (< 2500 g)	139 (96.50%)	140 (97.20%)	-	1.000
Apgar 1 min	7 (6–8)	7 (6–7)	-3.220	0.001
Apgar 5 min	8 (7–8)	8 (7-8.75)	-0.640	0.522
ARDS	65 (45.10%)	60 (41.70%)	0.353	0.552
Sepsis	11 (7.60%)	45 (31.3%)	25.626	< 0.001
Hyperbilirubinemia	103 (71.50%)	80 (55.6%)	7.929	0.005
Asphyxia	67 (46.50%)	52 (36.10%)	3.222	0.073
PDA	31 (21.50%)	32 (22.20%)	0.020	0.887
Pneumonia	51 (35.40%)	50 (34.70%)	0.015	0.902
**Feeding factors**				
Age of first feeding (days)	3 (2–3)	3 (2–3)	-0.924	0.355
HM proportion				
Non-HM (0%)	73 (50.69%)	95 (65.97%)	7.106	0.029
Low-HM (< 50%)	23 (15.97%)	14 (9.70%)		
High-HM (≥ 50%)	48 (33.33%)	35 (24.31%)		
Probiotics	21 (14.60%)	22 (15.30%)	0.027	0.869

Abbreviations: GDM, gestational diabetes mellitus; SAG: small for gestational age; LBW: low birth weight infant; ARDS, respiratory distress syndrome; PDA, patent ductus arteriosus; HMF: human milk feeding; HM: human milk

**Fig. 2:**
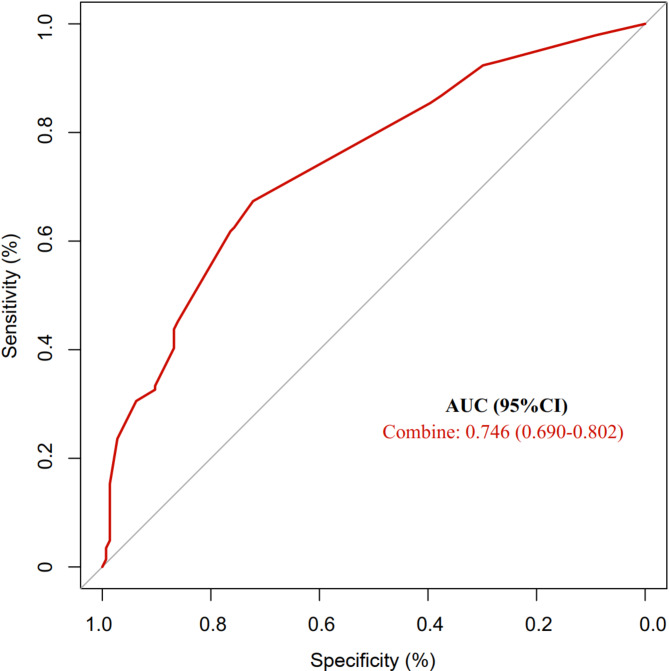
ROC curve for predicting influence factors of necrotizing enterocolitis. Figure Legends ROC, Receiver operating characteristic; AUC, areas under the receiver operating characteristic (ROC) curve; AUC = 0.746, SE = 0.029, *P* < 0.001, 95% CI:0.690–0.802;

A univariate analysis was performed to evaluate the correlation between putative risk variables and NEC before the diagnosis (see Table 2). The univariate analysis indicated significant variables, which were then analyzed using multivariate logistic regression. The research demonstrated that SGA, newborn sepsis, and non-HM feeding were identified as independent risk factors for NEC in premature babies, while hyperbilirubinemia was a protective factor for NEC in preterm infants (see Table 3). After adjusting for relevant confounders, the OR values and 95% CI (Confidence interval) were 0.347(0.150 to 0.799) and 0.480(0.264 to 0.871) for low and high HM feeding compared with non-HM feeding, respectively, suggesting that the risk of NEC was lower for both low and high HM feeding than non-HM feeding. The results were statistically significant (*p* < 0.05). In addition, we also verified the reliability of the results. The Hosmer-Lemeshow test showed no significant difference (c^2^ = 3.834, *p* = 0.872 > 0.05) between the expected frequency and the observed frequency obtained by the predicted probability, that is, the model fitting effect was good. The prediction model was built using multi-factor logistic regression’s acquired influencing elements. ROC analysis revealed that 95% CI was 0.690 to 0.802 and the AUC (area under the curve) was 0.746 (see Fig. 2). The model’s performance reveals that it is excellent at spotting newborn NEC.

**Table 3:** Multivariate regression analysis of risk factors for NEC in neonates

Variables	β	SE	Wald c^2^	*P*	OR	95%CI
SGA	1.054	0.326	10.461	0.001	2.870	1.515–5.436
Apgar 1 min	0.041	0.100	0.170	0.680	1.042	0.856–1.268
Hyperbilirubinemia	-0.619	0.273	5.159	0.023	0.538	0.316–0.919
Neonatal sepsis	1.735	0.385	20.352	< 0.001	5.671	2.668–12.054
HM proportion			9.599	0.008		
Low-HM (< 50%) ^a^	-1.060	0.426	6.177	0.013	0.347	0.150–0.799
High-HM (≥ 50%) ^a^	-0.735	0.304	5.837	0.016	0.480	0.264–0.871

Abbreviations: NEC, necrotizing enterocolitis; SGA: small for gestational age; SE: standard error; OR: odds ratios; 95%CI: 95% confidential interval. ^a^ Comparison with the non-HM feeding (0%)

## Discussion

Mostly affecting preterm babies, NEC is a devasting juvenile gastrointestinal disease that still ranks as the top cause of morbidity and death in the NICU [15]. Nearly one-third of neonates with NEC are fatal, and survivors are at high for poor long-term growth, neurodevelopment impairment, and other complications [16]. With the development of modern medicine and perinatal care, many strategies for preventing gastrointestinal diseases and related complications in preterm infants, such as vaccines and antimicrobial prophylaxis, have enabled the survival of many preterm infants. However, despite these advantages, an increased incidence of NEC has been observed [17, 18]. Despite advances in the understanding of the etiology and pathophysiology of NEC, there is a lack of tools for early definitive diagnosis, prevention strategies, and specific treatment modalities. Therefore, knowledge of risk factors in early prediction, identification, and timely treatment, is key to reducing NEC-related complications and fatalities. In this retrospective study, we examined the neonatal outcome of NEC and their underlying risk factors.

Our study found that SGA is a significant independent risk factor for NEC, aligning with existing research [19]. Previous studies have shown a higher incidence of NEC in preterm SGA neonates or suggested a similar trend. In our study, we also found smaller GA is positively associated with the probability of NEC. Some studies have shown that NEC exhibits a GA susceptibility, with a higher prevalence in infants at 30–32 weeks of gestation, possibly due to intestinal bacteria colonization, microcirculation perfusion, and physiological immaturity of the gastrointestinal tract [11, 20]. Meanwhile, before the onset of NEC, the intestinal flora has changed, and toll-like receptor 4 (TLR4) in the intestine can be recognized by lipopolysaccharide, leading to increased apoptosis. Hypoxic-ischemic injury, caused by reperfusion, along with increased proinflammatory factors, likely contributes to NEC onset. Additionally, gastrointestinal immaturity plays a role, as most NEC cases in our study involved preterm infants. The main reason is that premature intestinal tissue is undeveloped, mesenteric microvascular tension is increased, and the tight junction is immature, which may lead to intestinal proinflammatory signal transduction and bacterial translocation, which may increase susceptibility to NEC.

Our study suggests that neonatal sepsis is the most important predictor among the three risk factors (*p* < 0.05), with an OR of 5.671(95% CI: 2.668–12.054) for the occurrence of NEC. A recent study suggested a NEC incidence rate of 34-57% among neonates with sepsis [21, 22]. Stafford et al. [23] concluded that a significant correlation between early-onset invasive Group B streptococcus (EO-GBS) disease and NEC, with a more than five-fold increase in NEC risk among newborns of GBS-positive mothers. Garg et al. demonstrated that 50 of 209 neonates with sepsis developed NEC [24]. These findings reinforce the critical link between sepsis and NEC development. Previous studies found that the intestinal flora of neonates has changed before the onset of NEC, which is manifested as the reduction of bacterial diversity and alteration of intestinal flora [11]. Intestinal pathogenic bacteria invade the gastrointestinal tract, which can cause a systemic inflammatory response, and then lead to the development of NEC. Therefore, neonates with sepsis need early treatment to reduce the incidence of NEC.

Neonatal hyperbilirubinemia is a prevalent condition in newborns, noted for its characteristic jaundice symptom, which arises from an abnormal bilirubin metabolism. This condition can potentially progress to severe complications such as neonatal bilirubin encephalopathy and cerebral palsy [25]. Research indicates that over 80% of preterm infants suffer from hyperbilirubinemia. Bilirubin possesses antioxidant properties, enabling it to neutralize free radicals within the body and act as a plasma free radical scavenger against oxidative damage [26]. In cases of NEC among preterm infants, intestinal ischemia, hypoxia, and reperfusion injury generate an excessive amount of oxygen-free radicals. Elevated bilirubin levels can expedite the elimination of these free radicals, consequently reducing the onset of NEC. Our study further identified that hyperbilirubinemia functions as a protective factor against NEC.

Our study suggests that non-HM feeding is a significant risk factor for NEC in preterm infants, and the feeding pattern is closely related to the incidence of NEC. HM feeding is crucial in preventing NEC in preterm infants, as exclusively breastfed infants have a significantly lower risk compared to formula-fed infants. This could be attributed to the beneficial effects of protective components found in breast milk on preterm infants [27]. Studies have found that HM feeding may reduce the risk of NEC by modulating the gut microbiota [28]. Breastfeeding promotes the development of a rich and balanced microbiota, supporting the specific growth of beneficial probiotics, such as bifidobacteria and lactobacilli, which have been shown to have protective properties for the neonatal gut [29, 30]. In contrast, formula-fed infants are more likely to have colonization of non-beneficial bacteria such as Escherichia coli and Clostridium difficile in their intestines [31]. The microbiota of formula-fed infants is more susceptible to environmental influences, and formula feeding may promote excessive growth of gut bacteria and mucosal displacement, contributing to the development of NEC [32]. The differences in gut microbiota between breastfed and formula-fed infants primarily stem from human milk oligosaccharides (HMOs). HMOs, transferred to the infant through breast milk, selectively stimulate the proliferation or activity of beneficial bacteria, such as bifidobacteria and lactobacilli. They also inhibit the TLR4 and NLRP3 signaling pathways, reducing the production of inflammatory cytokines such as IL-1β, TNF-α, and IL-6, thereby lowering the incidence of NEC [33]. Therefore, advocating for HM feeding is of utmost importance.

Numerous investigations have looked at how HM exposure relates to NEC risk. Meinzen-Derr et al. [34]examined 1272 LBW babies from October 1999 to August 2001 and observed a dose-dependent relationship between HM consumption and lowered risk of NEC or mortality. In the first 14 days of life, Sisk et al. [35] also found that enteral feeding with at least 50% HM was linked with a sixfold drop in NEC chances. Cacho et al. ‘s study showed that more than 50% enteral HM feeding is required to ensure the protective effect of NEC [36]. These findings highlight that an HM proportion exceeding 50% in total enteral feeding is crucial for gastrointestinal immune regulation, particularly in very LBW infants. Our study found that compared with non-HM feeding, low HM feeding, and high HM feeding were an independent protective factor for NEC. Preterm infants with low and high HM feeding had a 65.3%and 52.0% lower risk of developing NEC, respectively. Moreover, studies have shown that higher HM feeding is more effective than lower HM feeding in preventing NEC [37]. However, our study found that, compared to high-HM feeding, low-HM feeding was associated with a greater reduction in NEC incidence. Although this result contradicts existing studies, we believe this may be due to the relatively small sample size of low-HM feeding in our study. In the control and NEC groups, the proportion of low-HM feeding was 15.97% and 9.7%, respectively. Due to the small sample size, this may have masked the dose-response relationship, thus affecting our interpretation of the results. Additionally, our study also shows that, compared to exclusive formula feeding, increasing the proportion of HM, even if below 50%, still provides some protective effect against NEC. This further emphasizes the importance of promoting HM feeding, especially in preterm infants. Even if exclusive breastfeeding is not feasible, increasing the proportion of HM remains an effective measure to prevent NEC.

This study has several limitations: (i) Its retrospective design may introduce information bias. (ii) Being a single-center research with a quite limited sample size, some significant group differences might have escaped notice. (iii) Due to the relatively small sample size of infants receiving low HM feeding, the dose-response relationship between HM feeding and NEC incidence may not have been fully captured. Future studies with larger sample sizes could help clarify this relationship and provide more robust evidence.

## Conclusion

In summary, the general occurrence of NEC is progressively rising, mostly in premature newborns. SGA, newborn infection, and non-HM feeding were shown to be distinct risk factors for NEC. One possible protective factor against NEC is newborn hyperbilirubinemia. Additionally, our findings indicate that low-HM feeding before the diagnosis of NEC and high-HM feeding, compared to non-HM feeding, were associated with a decreased risk of NEC and exhibited a notable protective impact. In the future, we must adopt a more proactive approach toward the treatment and care of SGA infants, children with sepsis, and non-HM feeding infants. Additionally, we must diligently monitor and take preventive measures to detect NEC at its earliest stage.

## Electronic supplementary material

Below is the link to the electronic supplementary material.

Supplementary Material 1

## Data Availability

The datasets generated during and/or analyzed during the current study are available from the corresponding author upon reasonable request.

## Abbreviations

AUC: Area under the curve
BW: Birth weight
CI: Confidence interval
EO-GBS: Early-onset invasive Group B streptococcus
EOS: Early-onset sepsis
GA: Gestational age
HM: Human milk
HMO: Human milk oligosaccharide
IQR: Interquartile percentile
LBW: Low birth weight
LOS: Late-onset sepsis
NICU: Neonatal intensive care unit
NEC: Necrotizing enterocolitis
OR: Odds ratios
ROC: Receiver operating characteristic
SGA: Small for gestational age
TLR4: Toll-like receptor 4
