# Supplementary material for: Analysis of risk factors and establishment of predictive models for neonatal necrotizing enterocolitis: a retrospective study
Source: Ital J Pediatr. 2025 Mar 14;51:80. doi: 10.1186/s13052-025-01930-y (PMC11909941; doi:10.1186/s13052-025-01930-y)
Supplement: Supplementary file 1 — Supplementary Material 1 [file 13052_2025_1930_MOESM1_ESM.docx]

Editor in Chief

Thank you for your letter dated February 18th. Thank you so much for having our manuscript (ITJP-D-24-00692) entitled “Analysis of risk factors and establishment of predictive models for neonatal necrotizing enterocolitis: A retrospective study” reviewed in a timely and professional manner. And thanks for giving us an opportunity to improve the manuscript further. We also deeply appreciate the critical review of the manuscript with thoughtful and constructive comments provided by the reviewers, based on which we have revised the manuscript. At the same time, according to your suggestions, we have improved the format of the manuscript.

While the changes made in the revised manuscript are highlighted in yellow, our point-by-point response to the comments are included at the bottom of this letter.

We hope this revised manuscript has addressed your concerns. Please let me know if there are any other suggestions, we would like very much to modify them and we really appreciate your help.

Once again, thank you very much for your suggestions. We look forward to your decision.

Sincerely,

Yanwei Su, M.D.

School of Nursing

Tongji Medical College

Huazhong University of Science and Technology

Wuhan, Hubei, 430030, China

Phone: (+86) 027-83692658

FAX: (+86) 027-83692658

Email: yanweisu@hust.edu.cn

**Responses to Reviewer #1:**

Dear reviewer:

Thanks a lot for the insightful comments on our manuscript. We have studied the comments very carefully and revised the manuscript. All the revised portions are highlighted in yellow and our point-by-point response to the comments is included at the bottom of this letter.

Once again, thank you very much for giving us suggestions for improvement. We look forward to meeting your approval.

Sincerely,

Yanwei Su, M.D.

School of Nursing

Tongji Medical College

Huazhong University of Science and Technology

Wuhan, Hubei, 430030, China

Phone: (+86) 027-83692658

FAX: (+86) 027-83692658

Email: yanweisu@hust.edu.cn

**Responses to the specific comments:**

**1 Comment:** Line 59: you write "neonatal hypercholesterolemia", but you probably means neonatal hyperbilirubinemia.
**Response: Abstract Line 59:** Thank you for your careful review and for pointing out this error. We sincerely apologize for the oversight, as the term "neonatal hypercholesterolemia" was incorrectly used. The correct term should be "neonatal hyperbilirubinemia," which refers to elevated bilirubin levels in neonates, leading to jaundice. We have corrected this in our manuscript to reflect the correct terminology. Thank you again for your valuable feedback.

**2 Comment:** Line 123: You write "the collected data were categorized into four main areas" but are described only 3 areas.
**Response: Data collection lines 125-126:** Thank you for pointing out this inconsistency. Due to an oversight on our part, we mentioned four main areas for data categorization but only described three in the original manuscript. We have now corrected this in the revised manuscript by adding the missing area in the data collection section. The updated manuscript now accurately reflects the original intention of categorizing the data into four areas. All the revised portions are highlighted in yellow and we hope to get your approval.

**Responses to Reviewer #2:**

Dear reviewer:

Thanks a lot for the insightful comments on our manuscript. We have studied the comments very carefully and revised the manuscript. All the revised portions are highlighted in yellow and our point-by-point response to the comments is included at the bottom of this letter.

Once again, thank you very much for giving us suggestions for improvement. We look forward to meeting your approval.

Sincerely,

Yanwei Su, M.D.

School of Nursing

Tongji Medical College

Huazhong University of Science and Technology

Wuhan, Hubei, 430030, China

Phone: (+86) 027-83692658

FAX: (+86) 027-83692658

Email: yanweisu@hust.edu.cn

**Responses to the overall suggestions:**

Congratulations to the authors
the work is well done, statistically robust and well presented. Although it explores a topic without providing particularly new data on the risk factors and predisposing factors for nec, it nevertheless presents and reiterates with the force of significant numbers the presence of some risk conditions useful for improving the management and prevention of this fearful neonatal pathology.
Some major and minor critical points, however, deserve to be clarified to make the work eligible for publication.

**Response:** Thank you for your valuable comments and suggestions on our manuscript. We really appreciate your recognition of our work. We know that while our study may not provide particularly new data on risk and predisposition factors for NEC, it does help highlight and confirm these factors, which we believe will help improve management and prevention strategies for neonatal care. Thank you for your constructive comments and we will further clarify and refine the manuscript in response to the major and minor points you have raised. All the revised portions are highlighted in yellow and we hope to get your approval.

**Responses to the specific comments:**

**1 Comment:** hyperbilirubinemia is presented as risk factor among results, but it is presented as a protective one in the discussion section, being thus misleading for the reader. Please clarify and remodulate this point
hypecholesterolemia, conversely, is presented in the abstract, but not more mentioned throughout the text, please clarify
**Response: Abstract Line 59, Result lines 192-193:** Thank you for your careful review and valuable feedback. Due to an oversight on our part, we mistakenly described hyperbilirubinemia as a risk factor in the results section, while in the discussion, we correctly identified it as a protective factor for neonatal NEC. We have now corrected the description in the results section to classify hyperbilirubinemia as a protective factor. Regarding hypercholesterolemia, we apologize for the confusion caused by the incorrect use of this term in the abstract. The correct term should be "neonatal hyperbilirubinemia", referring to elevated bilirubin levels in neonates, leading to jaundice. We have revised the abstract to reflect the correct terminology and made corresponding adjustments throughout the manuscript to ensure consistency. All the revised portions are highlighted in yellow and we hope to get your approval. Once again, thank you for your valuable feedback, which has greatly helped improve the clarity and accuracy of our manuscript.

**2 Comment:** In the data collection clarify what low-HM and high-HM consist of, as made later in the text

probiotics use is quite aspecific, also in this case the authors should provide a specification, as different species may have significantly different roles and effects for health

**Response:** **Data collection, lines 134-139:** Thank you for your constructive suggestions. In response to your feedback, we have made the necessary revisions to the manuscript and added the relevant definitions. In the data collection section, we have clarified the definitions of "low-HM" and "high-HM". Infants were grouped based on the proportion of HM intake during hospitalization relative to their total enteral feeding (0%，<50%, ≥50%). Infants with ≥50% HM intake were categorized into the high-HM group, while those with <50% HM intake were categorized into the low-HM group. Infants receiving no HM or exclusively formula-fed were classified into the non-HM group. Furthermore, regarding the use of probiotics, we agree with your point that the specific strains of probiotics are crucial, as different species can have distinct health effects. We have revised the manuscript to specify that the probiotics used in our study were Bifidobacterium and Lactobacillus. We believe these changes will enhance the accuracy and transparency of our manuscript. All the revised portions are highlighted in yellow and we hope to get your approval.

**3 Comment:** Results

"Of these, 85 (59.03%) males and 59 (40.97%) females in the NEC cohort and control group, respectively" please clarify this sentence as "respectively" appears misleading, since the proportion between genders in the two groups is the same, as confirmed also in table 1

**Response:** **Results, lines 170-172:** Thank you for your insightful comments. We sincerely apologize for the confusion caused by the use of "respectively" in this sentence, as the proportions of males and females are the same in both the NEC group and the control group, as shown in Table 1. For clarity, we have revised the sentence as follows: "In both the NEC cohort and the control group, the gender proportions were the same, with males accounting for 59.03% and females for 40.97%". We hope this revision addresses your concern and improves the clarity of the manuscript. Once again, thank you for your valuable feedback.

**4 Comment:** Discussion

abut the development of modern medicine and perinatal medicine the authors should consider and mention other efficient strategies of prevention of gastrointestinal diseases and/or related complications, such as vaccines and antimicrobial (including antifungal) prophylaxis. In regard of this aspect, please cite and include within reference list the following studies:

M. Giuffrè, C. Lo Verso, G. Serra, G. Moceri, M. Cimador, G. Corsello; Study Group of Neonatal Infectious Diseases Affiliated to the Italian Society of Neonatology.Portal Vein Thrombosis in a Preterm Newborn with Mutation of the MTHFR and PAI-1 Genes and Sepsis by Candida parapsilosis. American Journal of Perinatology. 2016 Sep;33(11):1099-103.

Acute pancreatitis in children and rotavirus infection. Description of a case and minireview. S. Giordano, G. Serra, P. Dones, M. Di Gangi, M.C. Failla, C. Iaria, F. Ricciardi, L.M. Pernice, D. Pantaleo, A. Cascio. New Microbiologica. 2013 Jan;36(1):97-101.

**Response:** **Discussion, lines 209-213:** Thank you for your valuable advice. We share your view that effective strategies to prevent gastrointestinal diseases and related complications, including vaccine and antimicrobial prophylaxis, are an important aspect of modern perinatal medicine. We have added a discussion of these prevention strategies to the revised manuscript, highlighting their relevance in the prevention of neonatal gastrointestinal disease. In addition, we cited the two studies you mentioned and included them in the list of references. All the revised portions are highlighted in yellow and we hope to get your approval.

**5 Comment:** About the high rates of morbidity and mortality related with NEC also in high income countries, please consider and add among the bibliographic entries the following paper:

Necrotizing enterocolitis in the preterm: newborns medical and nutritional Management in a Single-Center Study. Giovanni Savarino, Maurizio Carta, Marcello Cimador, Antonio Corsello, Mario Giuffrè, Ingrid Anne Mandy Schierz, Gregorio Serra and Giovanni Corsello. Ital J Pediatr 2021 Nov;47: 226.

**Response:** **Introduction, lines 81-85:** Thank you for your valuable suggestion. In response to your recommendation, we have included this paper in our list of references. This study provides important insights into the clinical characteristics, treatment approaches, and nutritional management of NEC in preterm infants, highlighting the high morbidity and mortality associated with NEC, even in high-income countries. We believe that incorporating this reference strengthens the comprehensiveness of our discussion on the morbidity and mortality rates related to NEC. All the revised portions are highlighted in yellow and we hope to get your approval.

**6 Comment:** line 216, change the full stop with the comma after "changed", and write "And" in lowercase

change "an NEC" with "a NEC incidence rate"

**Response:** **Discussion, line 226, line 236:** Thank you for your thoughtful suggestions. We have made the following revisions to the manuscript. In discussion, line 226, as per your recommendation, we changed the full stop after "changed" to a comma and made "and" lowercase to ensure the sentence flows more smoothly. In discussion, line 236, we also revised "an NEC" to "a NEC incidence rate" to provide greater clarity and accuracy in describing the incidence of NEC. We have carefully reviewed the manuscript, and we appreciate your attention to detail. We believe these changes enhance the quality of our manuscript. Once again, thank you for your valuable feedback.

**7 Comment:** neonatal sepsis was the most important predictor of NEC among the three risk factors, please specify for better clarity of the reader
**Response: Discussion lines 234-235:** Thank you for your valuable feedback. In response to your comment, we have added the following clarification in the discussion section to ensure better clarity for the reader: "Our study suggests that neonatal sepsis is the most important predictor among the three risk factors (*p* < 0.05), with an OR of 5.671 (95% CI: 2.668-12.054) for the occurrence of NEC." We believe this addition provides clearer context for the reader, emphasizing the statistical significance of neonatal sepsis as a predictor of NEC. Thank you again for your helpful suggestion.

**8 Comment:** the role of HM in modulating the intestinal microbiota should be better explained in terms of beneficial effects due to the balance between lactobacilli and bifidobacteria species

**Response: Discussion lines 261-275：**Thank you for your valuable suggestion. In response, we have added the discussion in the manuscript to better explain the beneficial effects of HM in modulating the gut microbiota, specifically focusing on the balance between lactobacilli and bifidobacteria species. We have added the following content to our manuscript: "Studies have found that HM feeding may reduce the risk of NEC by modulating the gut microbiota [1]. Breastfeeding promotes the development of a rich and balanced microbiota, supporting the specific growth of beneficial probiotics, such as bifidobacteria and lactobacilli, which have been shown to have protective properties for the neonatal gut [2, 3]. In contrast, formula-fed infants are more likely to have colonization of non-beneficial bacteria such as Escherichia coli and Clostridium difficile in their intestines [4]. The microbiota of formula-fed infants is more susceptible to environmental influences, and formula feeding may promote excessive growth of gut bacteria and mucosal displacement, contributing to the development of NEC [5]. The differences in gut microbiota between breastfed and formula-fed infants primarily stem from human milk oligosaccharides (HMOs). HMOs, transferred to the infant through breast milk, selectively stimulate the proliferation or activity of beneficial bacteria, such as bifidobacteria and lactobacilli. They also inhibit the TLR4 and NLRP3 signaling pathways, reducing the production of inflammatory cytokines such as IL-1β, TNF-α, and IL-6, thereby lowering the incidence of NEC [6]. Therefore, advocating for HM feeding is of utmost importance." We believe this expanded discussion provides a clearer understanding of the beneficial effects of HM on the gut microbiota and its role in preventing NEC. All the revised portions are highlighted in yellow and we hope to get your approval.

[1] Pammi M, Cope J, Tarr PI, Warner BB, Morrow AL, Mai V, Gregory KE, Kroll JS, McMurtry V, Ferris MJ et al: Intestinal dysbiosis in preterm infants preceding necrotizing enterocolitis: a systematic review and meta-analysis. Microbiome 2017, 5(1):31.

[2] Pantazi AC, Balasa AL, Mihai CM, Chisnoiu T, Lupu VV, Kassim MAK, Mihai L, Frecus CE, Chirila SI, Lupu A et al: Development of Gut Microbiota in the First 1000 Days after Birth and Potential Interventions. Nutrients 2023, 15(16).

[3] Yatsunenko T, Rey FE, Manary MJ, Trehan I, Dominguez-Bello MG, Contreras M, Magris M, Hidalgo G, Baldassano RN, Anokhin AP et al: Human gut microbiome viewed across age and geography. Nature 2012, 486(7402):222-227.

[4] Zanella A, Silveira RC, Roesch LFW, Corso AL, Dobbler PT, Mai V, Procianoy RS: Influence of own mother's milk and different proportions of formula on intestinal microbiota of very preterm newborns. Plos One 2019, 14(5).

[5] Gregory KE, Samuel BS, Houghteling P, Shan G, Ausubel FM, Sadreyev RI, Walker WA: Influence of maternal breast milk ingestion on acquisition of the intestinal microbiome in preterm infants. Microbiome 2016, 4(1):68.

[6] Zhang H, Wang Y, Li S, Tang X, Liang R, Yang X: SOCS3 protects against neonatal necrotizing enterocolitis via suppressing NLRP3 and AIM2 inflammasome activation and p65 nuclear translocation. Mol Immunol 2020, 122:21-27.

**9 Comment:** We found that low HM feeding was associated with a greater reduction in the incidence of NEC: the explanation the authors provided should be better argued as the consideration "the proportion of HM in both the control and NEC groups in our research was less than 50%" seem poorly clear or at least incomplete

**Response: Discussion lines 286-297, limitation lines 300-303:** Thank you for your valuable comments. We sincerely apologize for any lack of clarity in our explanation regarding how low-HM feeding is associated with a greater reduction in NEC incidence. To address this issue, we have revised the manuscript to provide a clearer explanation. The revised content is as follows: Studies have shown that higher HM feeding is more effective than lower HM feeding in preventing NEC. However, our study found that, compared to high-HM feeding, low-HM feeding was associated with a greater reduction in NEC incidence. Although this result contradicts existing studies, we believe this may be due to the relatively small sample size of low-HM feeding in our study. In the control and NEC groups, the proportion of low-HM feeding was 15.97% and 9.7%, respectively. Due to the small sample size, this may have masked the dose-response relationship, thus affecting our interpretation of the results. We have included this clarification in the limitations section. Additionally, our study also shows that, compared to exclusive formula feeding, increasing the proportion of HM, even if below 50%, still provides some protective effect against NEC. This further emphasizes the importance of promoting HM feeding, especially in preterm infants. Even if exclusive breastfeeding is not feasible, increasing the proportion of HM remains an effective measure to prevent NEC. The changes are reflected in the discussion section, lines 286-297, and the limitation section, lines 300-303, and all modifications are highlighted in yellow. We hope these revisions provide a clearer explanation of the results and further strengthen the manuscript's integrity. Once again, thank you for your valuable feedback, which has greatly contributed to improving the quality of our paper.

**10 Comment:** table 2
in vitro fertilization
premature rupture of membranes
**Response:** **table 2:** Thank you for your helpful comment. According to your suggestion, we have made the necessary revisions in Table 2. "Vitro fertilization" has been updated to "in vitro fertilization" and "rupture of membranes" has been updated to "premature rupture of membranes" for clarity and accuracy. We appreciate your attention to detail, and we believe these changes improve the manuscript. Thank you again for your valuable feedback.

**11 Comment:** Figure 1
17 had hospital stays of less than 7 days
13 did not start feeding before the diagnosis of NEC

**Response: Figure 1:** Thank you for your constructive comments. Following your recommendation, we have revised the relevant content in Figure 1. We appreciate your attention to detail and believe these changes will help readers better understand the content of Figure 1. Once again, thank you for your valuable feedback.

**12 Comment:** table 2
neonatal...neonatal avoid such repetitions
**Response: table 2:** Thank you for your insightful comment. According to your advice, we have removed the redundant use of "neonatal" in Table 2 to improve clarity and avoid repetition. We appreciate your attention to detail and believe this revision enhances the manuscript. Thank you again for your valuable feedback.

Once again, thanks so much for all the suggestions! We hope to get your approval.
